# A Method to Estimate the Chronic Health Impact of Air Pollutants in U.S. Residences

**DOI:** 10.1289/ehp.1104035

**Published:** 2011-11-17

**Authors:** Jennifer M. Logue, Phillip N. Price, Max H. Sherman, Brett C. Singer

**Affiliations:** Environmental Energy Technologies Division, Lawrence Berkeley National Lab, Berkeley, California, USA

**Keywords:** air toxics, criteria pollutants, DALYs, exposure, impact assessment, indoor air pollutants, indoor air quality

## Abstract

Background: Indoor air pollutants (IAPs) cause multiple health impacts. Prioritizing mitigation options that differentially affect individual pollutants and comparing IAPs with other environmental health hazards require a common metric of harm.

Objectives: Our objective was to demonstrate a methodology to quantify and compare health impacts from IAPs. The methodology is needed to assess population health impacts of large-scale initiatives—including energy efficiency upgrades and ventilation standards—that affect indoor air quality (IAQ).

Methods: Available disease incidence and disease impact models for specific pollutant–disease combinations were synthesized with data on measured concentrations to estimate the chronic heath impact, in disability-adjusted life-years (DALYs) lost, due to inhalation of a subset of IAPs in U.S. residences. Model results were compared with independent estimates of DALYs lost due to disease.

Results: Particulate matter ≤ 2.5 μm in aerodynamic diameter (PM_2.5_), acrolein, and formaldehyde accounted for the vast majority of DALY losses caused by IAPs considered in this analysis, with impacts on par or greater than estimates for secondhand tobacco smoke and radon. Confidence intervals of DALYs lost derived from epidemiology-based response functions are tighter than those derived from toxicology-based, interspecies extrapolations. Statistics on disease incidence in the United States indicate that the upper-bound confidence interval for aggregate IAP harm is implausibly high.

Conclusions: The approach demonstrated in this study may be used to assess regional and national initiatives that affect IAQ at the population level. Cumulative health impacts from inhalation in U.S. residences of the IAPs assessed in this study are estimated at 400–1,100 DALYs lost annually per 100,000 persons.

Air pollutant concentrations in many homes exceed health-based standards for chronic and acute exposures (Logue et al. 2011). On average, Americans spend > 65% of their time in residences (Klepeis et al. 2001), and numerous studies have noted the importance of the indoor environment to cumulative air pollutant intake (Samet 1993; Weisel et al. 2005). Impact assessment methods have been applied to estimate aggregate chronic health impacts for outdoor air pollution [Muller and Mendelsohn 2007; U.S. Environmental Protection Agency (EPA) 1999] and from pollutant inhalation in office buildings (Fisk et al. 2011). Yet, to our knowledge, no study has yet considered both disease incidence and severity to assess aggregate health impacts of air pollutant inhalation in residences.

Air pollutants known to be hazardous based on epidemiological and toxicological information include the “criteria pollutants” specified in the 1970 Clean Air Act Amendments (CAAA 1970) and hazardous air pollutants (HAPs) specified in the 1990 CAAA (1990). In addition, concern is growing that some bioaccumulating semivolatile organic compounds and ultrafine particles—both ubiquitously present in residences—may cause substantial adverse health effects at typical environmental levels. However, the current toxicological data is insufficient to quantify that impact. The U.S. EPA and the California EPA (CalEPA) each publish health standards or guidelines for long-term exposure concentrations to protect against cancer and noncancer chronic effects. The hazard associated with residential air pollutant exposure can be quantified as the percentage of homes that exceed specified noncancer standards or as the incremental risk of cancer incidence across the population. These methods consider only disease potential for noncancer end points and disease incidence for cancer; they do not incorporate disease severity. Quantitatively comparing the effects of individual residential indoor air pollutants (IAPs) and comparing their estimated cumulative health impact with that of other environmental hazards requires a single metric that includes both disease incidence and severity. A comprehensive metric will facilitate the evaluation of residential indoor air quality (IAQ) interventions, including source control measures and ventilation.

Epidemiological and toxicological research has contributed to the development of tools to bridge the gap from measured pollutant exposure levels to disease incidence, and from disease incidence to health costs in disability-adjusted life-years (DALYs) lost. Concentration–response (C-R) relationships have been quantified for criteria pollutants. The U.S. EPA aggregated several of these disease incidence models for use in the cost-benefit analysis of the Clean Air Act (U.S. EPA 1999). Several studies have estimated the health impact per incidence of specific diseases (Hong et al. 2010; Lvovsky et al. 2000; Melse et al. 2010). Huijbregts et al. (2005) published cumulative impact and effect factors for exposure to air pollutants, including air toxics and ozone. These models provide the basis for performing a human health impact assessment for inhalation of IAPs.

In this study we combined disease incidence and DALY-based health impact models to develop a methodology for estimating the population-average health costs due to chronic inhalation of a broad suite of air pollutants in U.S. residences. We first analyzed published data to calculate mean exposure concentrations and estimated age-dependent inhalation intakes. We used disease incidence and disease impact models to predict pollutant-specific impacts and total DALY-based health costs to identify the residential IAPs that have the greatest impact on health in the United States. As a check on the method, and the estimated aggregate impact of IAP, we compared our findings with independent estimates of DALY losses related to secondhand smoke (SHS) to diseases that could potentially result from air pollutant exposure and to all noncommunicable, nonpsychiatric diseases in the United States.

## Methods

*IAP intake*. To calculate pollutant inhalation in U.S. residences, we used a data compilation described by Logue et al. (2011) that includes summary statistics from 77 studies reporting residential air pollutant concentration measurements in the United States and other countries with similar lifestyles. The aggregate data were used to calculate concentrations relevant to assessing chronic residential exposures to 267 chemical air pollutants. Seventy of the pollutants had sufficient toxicological and epidemiological data to calculate chronic health impact using the methodology described below and were included in this study (Table 1). Our analysis did not extend to contaminants from biological sources such as molds and allergens. We thus refer to the suite of pollutants considered as “nonbiological.”

**Table 1:** Pollutants included in analysis and assumed population-average concentrations (µg/m^3^).

Pollutant	Concentration
1,1,2,2-Tetrachloroethane	0.42
1,1,2-Trichloroethane	0.46
1,1-Dichloroethene	1.2
1,2-Dibromoethane	0.14
1,2-Dichloroethane	0.34
1,3-Butadiene	0.46
1,4-Dichlorobenzene	50
2-Butoxyethanol	2.6
2-Ethylhexanol	3.7
2-Ethoxyethanol	0.43
2-Methoxyethanol	0.12
Acetaldehyde	22
Acrolein	2.3
Acrylonitrile	0.27
Ammonia	28
Arsenic	9.8 × 10^–4^
Atrazine	5.9 × 10^–4^
Benzaldehyde	2.5
Benzene	2.5
Benzo[*a*]pyrene	9.1 × 10^–5^
Benzyl chloride	0.5
Beryllium	1.6 × 10^–6^
Bis(2-ethylhexyl) phthalate	0.14
Bromodichloromethane	0.49
Bromoform	0.39
Cadmium	2.6 × 10^–3^
Carbon disulfide	0.34
CO	810
Carbon tetrachloride	0.68
Chlorobenzene	0.68
Chloroethane	0.26
Chloroform	1.5
Chloromethane	1.8
Chromium	2.2 × 10^–3^
Crotonaldehyde	4.7
Pollutant	Concentration
Cyclohexane	5.2
Di(2-ethylhexyl) adipate	1.6 × 10^–2^
Dibenzo[*a*,*c*+*a*,*h*]anthracene	1.4 × 10^–5^
Dibromochloromethane	0.44
*d*-Limonine	23
Ethanol	860
Ethylbenzene	3.9
Formaldehyde	69
Hexachlorobutadiene	1.7
Hexane	7.3
Isopropylbenzene	0.4
Manganese	3.3 × 10^–3^
Methyl ethyl ketone	7.4
Mercury	1.6 × 10^–4^
Methyl methacrylate	0.27
Methylene chloride	8.2
Methyl isobutyl ketone	1.2
Methyl *tert*-butyl ether	12
Naphthalene	1.2
NO_2_	13.1
*o*-Phenylphenol	0.13
Ozone	17.2
Pentachlorophenol	2.9 × 10^–3^
PM_2.5_	15.9
Styrene	5.9
SO_2_	2.9
Tetrachloroethene	1.7
Tetrahydrofuran	15
Toluene	2.3
Trichloroethene	0.16
Vinyl chloride	1.7
Xylene, *o*	8.2
Xylene, *m*/*p*	9.7
Xylenes	7.4

*Determining annual population health impact.* The annual health impact of residential IAPs was calculated by considering the total intake in residences as an increment adding to intake in other environments. The increment was calculated by considering in-home inhalation of air containing the population-mean exposure concentrations relative to the theoretical case of the population inhaling residential air containing no pollutants.

The DALY metric allows quantification and comparison of the health costs from varied disease end points that can result from various pollutants. As a measure of equivalent years of life lost (YLL) due to illness or disease, DALY loss quantifies overall disease costs (impacts) due to both mortality and morbidity. DALY losses include YLL due to premature mortality and equivalent YLL due to reduced health or disability (YLD). For each disease, the DALYs lost per incidence are calculated as follows:

DALY_disease_ = YLL_disease_ + YLD_disease_. [1]

The equivalent life-years lost to reduced health are weighted from 0 to 1 based on the severity of the disease. For example, a 5-year illness that reduces quality of life to 4/5 that of a healthy year is valued at 1 DALY lost.

Several authors have determined the DALYs lost per incidence of specific diseases using the preeminent work of Murray and Lopez (1996a, 1996b) [Huijbregts et al. 2005; Lvovsky et al. 2000; World Health Organization (WHO) 2009]. Multiplying disease incidence by a “DALY factor” yields total DALYs lost per disease incidence:

DALYs = (∂DALYs/∂disease incidence) × disease incidence. [2]

Equation 2 uses a partial derivative in recognition that DALY losses are incrementally affected by causes other than disease. The total burden of disease in a community can be calculated as the aggregate, across all diseases, of DALY factors multiplied by disease incidence rates.

Our analysis used two approaches to calculate DALY losses from estimated exposure concentrations. For criteria pollutants [ozone, nitrogen dioxide (NO_2_), particulate matter ≤ 2.5 μm in aerodynamic diameter (PM_2.5_), sulfur dioxide (SO_2_), and carbon monoxide (CO)] we used an intake–incidence–DALY (IND) method that uses epidemiology-based C-R functions to quantify disease incidence rates; these are combined with estimates of DALY losses per disease incidence reported in the literature. For noncriteria pollutants we used an intake–DALY (ID) approach using the work of Huijbregts et al. (2005) to calculate the health impact associated with intake of noncriteria pollutants based on animal toxicity literature. The IND approach is preferred because it does not require interspecies extrapolations, which generally involve larger uncertainties than the epidemiologically based C-R functions. However, the IND approach can be used only for pollutants with information on C-R functions in humans. Ozone was the only pollutant for which both the IND and ID approaches could be applied.

Although the disease incidence relationships in the IND and ID approaches are accepted health impact models, they are nevertheless simplifications of populationwide responses to chronic inhalation exposure. Our approaches use linear (i.e., IND) and nearly linear (i.e., ID) disease incidence models without effect thresholds. For these types of disease incidence models, only the mean of the concentration distribution is needed to estimate population impact. Existence of a threshold concentration for disease incidence, or a strongly nonlinear disease-to-intake response, would necessitate accurate determination of the shape of the population intake distribution. A discussion of the impact of threshold effects on our DALY loss estimates is included in the “Discussion.” The potential impacts of nonlinear response functions are beyond the scope of the present study.

*The IND approach.* The first step of the IND method comprises the application of C-R functions to determine disease incidence. For almost all of the disease outcomes, the C-R function follows the formula:

ΔIncidence = – {*y*_0_ × [exp(–βΔ*C*_exposure_) – 1]} × population, [3]

where *y*_0_ is the baseline prevalence of illness per year, β is the coefficient of the concentration change, *C*_exposure_ is the exposure-related concentration, and population is the number of persons exposed. For each pollutant and outcome, *y*_0_ and β vary. Respiratory illness due to long-term NO_2_ intake requires a slightly different C-R functional form but still relies on a β with specified uncertainty.

When aggregating C-R functions and DALY factors, we tried to include all of the diseases with available established relationships between concentrations and disease incidence. We did not include diseases/outcomes that were negligible compared with the other diseases included. The health end points selected and DALY loss per incidence of disease are summarized in Table 2.

**Table 2:** Criteria pollutant C-R function outcomes and DALYs lost per incidence.

Pollutant	Outcome	β-Coefficient (95% CI)	*y*_0_	DALYs lost per incidence (95% CI)
PM_2.5_		Total mortality (Pope et al. 2002)		0.058 (0.002, 0.010)		7.4 × 10^–3^		1.4 (0.14, 14) (Pope 2007; Pope et al. 2002, 2009)
		Chronic bronchitis (Abbey et al. 1995)		0.091 (0.078, 0.105)		0.4 × 10^–3^		1.2 (0.12, 12) (Lvovsky et al. 2000; Melse et al. 2010)
		Nonfatal stroke (Brook et al. 2010)		0.025 (0.002, 0.048)		0.2 × 10^–3^		0 complications: 9.5 (9.25, 9.75)
								1 complication: 11.7 (11.1, 12.4)
								> 1 complication: 13.1 (12.2, 14.0) (Hong et al. 2010)
CO		Hospital admissions (Burnett et al. 1999)						4 × 10^–4^ (Lvovsky et al. 2000)
		Asthma		0.033 (0.016, 0.050)		1.8 × 10^–3^		
		Lung disease		0.025 (0.000, 0.057)		2.1 × 10^–3^		
		Dysrhythmias		0.058 (0.012, 0.102)		2.4 × 10^–3^		
		Heart failure		0.034 (0.002, 0.066)		3.4 × 10^–3^		
NO_2_		Hospital admissions (Burnett et al. 1999)						4 × 10^–4^ (Lvovsky et al. 2000)
		Respiratory issues		0.004 (0.000, 0.008)		9.5 × 10^–3^		
		Congestive heart failure		0.003 (0.001, 0.004)		3.4 × 10^–3^		
		Ischemic heart disease		0.003 (0.002, 0.004)		8.0 × 10^–3^		
		Respiratory illness, indicated by symptoms (Hasselblad et al. 1992)		0.028 (0.002, 0.053)		N/A		4 × 10^–4^ (Lvovsky et al. 2000)
Ozone		Mortality (Jerrett et al. 2010; Samet et al. 1997)		0.001 (0.000, 0.002)		7.7 × 10^–3^		1.0 (0.1, 10) (Levy et al. 2001; Lvovsky et al. 2000)
		Hospital admissions (Burnett et al. 1999)						4 × 10^–4^ (Lvovsky et al. 2000)
		Asthma		0.003 (0.001, 0.004)		1.8 × 10^–3^		
		Lung disease		0.003 (0.001, 0.005)		2.1 × 10^–3^		
		Respiratory infection		0.002 (0.001, 0.003)		5.8 × 10^–3^		
		Dysrhythmias		0.002 (0.000, 0.004)		2.4 × 10^–3^		
SO_2_		Hospital admissions (Burnett et al. 1999)		0.002 (0.000, 0.003)		8.0 × 10^–3^		4 × 10^–4^ (Lvovsky et al. 2000)
N/A, not applicable. *y*_0_ is the baseline prevalence of illness per year, and β is the coefficient of the concentration change used for inputs into Equation 3.								

Chronic PM_2.5_ exposure affects both the respiratory and cardiovascular systems. The three outcomes that we included were all-cause mortality, chronic bronchitis, and stroke. Pope et al. (2002) predicted incidence rates of all-cause mortality and the average YLL per unit increase in PM_2.5_ (Pope et al. 2009); we divided the former by the latter to get DALYs lost per incidence. The 95th percentile range of the DALYs lost per death was set to represent the span of values seen in the literature (Lvovsky et al. 2000; Pope et al. 2009). Recent studies have shown that chronic PM_2.5_ exposure can lead to heart disease and thickening of arterial walls (Künzli et al. 2004). The total impact of PM_2.5_ on cardiovascular health is not known. However, recent work by Miller et al. (2007) has shown associations between chronic PM_2.5_ and stroke, an outcome of heart disease, in women. The end point of nonfatal stroke was included in the analysis using the hazard ratios derived by Miller et al. (2007) for both men and women. This is likely an underestimation of the total impact of PM_2.5_ on heart disease. The DALYs lost per nonfatal stroke incidence were taken from Brook et al. (2010). The incidence of stroke predicted was split among 0, 1, and > 1 complications, and the percentage of stroke that resulted in death was determined based on the findings of Brook et al. (2010). Burnett et al. (1999) developed a C-R function for hospital admissions associated with long-term PM_2.5_ exposure. However, because the impact was negligible compared with the impact of mortality, chronic bronchitis, and stroke, we did not include this outcome. There is evidence that PM_2.5_ exposure is associated with other health outcomes, including diabetes and reduced lung function; however, these findings are relatively new and have not been included in this work.

For CO and SO_2_, the only outcomes relevant to chronic exposure appear to be hospital admissions. Chronic ozone and NO_2_ exposure have been associated with early death and respiratory illness, respectively. The input parameters into the C-R functions for these outcomes are the same as those used in the U.S. EPA cost–benefit analysis of the Clean Air Act (U.S. EPA 1999). For hospital admission and respiratory illness, we used the DALY loss/incidence values available in the literature. For ozone mortality, as with PM_2.5_, it is unclear how much life is lost because of early death. Values in the literature range from a few weeks to 10 years; we chose a large range of values to represent this uncertainty (Levy et al. 2001; Lvovsky et al. 2000).

The C-R functions are formulated to calculate the increment of disease incidence per increment of exposure concentration, not total disease incidence for a given exposure concentration. According to population-weighted demographics (Klepeis et al. 2001; U.S. Census Bureau 2010), summarized in Table 3, the “average” American spends 70% of the time in residences. The chronic exposure-relevant concentration contributed from indoor exposure was therefore set to 70% of the indoor concentration:

**Table 3:** Residential occupancy characteristics.

Age (years)	Percent of population	Cancer ADAF	Percent of day spent at home	Air intake (m^3^/day)
≤ 2		3		10		75		7
2–16		19		3		75		13
≥ 16		78		1		69		15
Population average		—		1.6		70		14.4
The percentage of the population in each of these age groups was determined from the U.S. Census Bureau (2010). The percentage of time each age groups spends at home was determined from the National Human Activity Pattern Study (Klepeis et al. 2001). The age-dependent inhalation rate was taken from U.S. EPA (2009).								

Δ*C*_exposure_ = 0.7*C*_indoors_. [4]

Incidence rates were combined with DALY factors to calculate total health impacts by pollutant (Equation 2). A Monte Carlo approach was used to calculate impacts by pollutant by sampling with replacement from the available distributions of DALY factors and β. We assumed that all DALY factor distributions are log-normal.

*The ID approach.* The ID approach extrapolated directly from indoor concentrations to total DALYs lost due to intake of specific pollutants. From this standpoint, it is convenient to rewrite Equation 2 as follows:

DALYs = (∂DALY/∂disease incidence) × (∂disease incidence/∂intake) × intake, [5]

where intake is the mass of pollutant that an individual inhales over a given time frame. Huijbregts et al. (2005) computed expected ranges of human impact for cancer and noncancer chronic effects of 1,192 substances, applying equal weightings for a year lost, independent of age (i.e., zero discounting). Using the values determined by Huijbregts et al. (2005), the DALYs lost for 1 year of breathing pollutant *i* is calculated using the following equations:

DALYs*_i_* = (∂DALY/∂intake) × intake, [6]

DALYs*_i_* = *C_i_* × *V* × [(∂DALY_cancer_/∂intake)*_i_* × ADAF + (∂DALY_noncancer_/∂intake)*_i_*], [7]

where ∂DALY/∂intake*_i_* are the cancer and noncancer mass intake-based DALY factors, *C_i_* is the indoor concentration, *V* is volume of air breathed in the residence each year, and ADAF is the age-dependent adjustment factor for cancer exposure as described below.

The age at which carcinogens are inhaled has an appreciable effect on total toxicity, and the U.S. EPA has developed ADAFs to calculate cancer health impact as a function of exposure age (U.S. EPA 2005). To align with U.S. EPA-recommended ADAFs, we considered three age groups: < 2, 2–16, and > 16 years of age (U.S. EPA 2005). A population-weighted average annual air intake volume and ADAF were calculated by combining age distribution of the U.S. population, age-specific inhalation rates, and time spent at home (Table 3).

Huijbregts et al. (2005) presented, for each chemical, both a central estimate (50th percentile value) and the estimated uncertainty of the DALY losses per mass intake of pollutant; uncertainty was assumed to be log-normal, characterized by a factor, *k_i_*, calculated as follows:

*k_i_* = (97.5th percentile/2.5th percentile)^0.5^, [8]

which includes the aggregated uncertainty of the rate of disease incidence as well as the uncertainty in the DALY losses per incidence of disease. We used a Monte Carlo approach to sample with replacement from uncertainty distributions of DALY factors derived from the central estimate of the DALY factor and the *k_i_* value, to determine the central estimate and 95% confidence interval (CI) for combined cancer and noncancer DALY losses for each pollutant. A Monte Carlo approach was also used to determine the total DALYs lost from all of the pollutants analyzed using the IND and ID methods.

Despite the availability of a DALY factor for bromomethane, DALY-based impacts are not presented for this compound because the limited available concentration data (New York State Department of Health 2006) appear more indicative of a local outdoor source than of general conditions in U.S. homes (Logue et al. 2011).

*Radon, SHS, and acute CO poisoning deaths.* The population-average DALYs lost to radon, SHS, and acute CO poisoning deaths were determined based on estimates of disease incidence from the literature. We included DALY loss estimates for these pollutants for two reasons: *a*) to compare health impacts calculated for a subset of SHS pollutants using the IND and ID methodologies to independent estimates of overall DALY losses associated with SHS exposure (as described below), and *b*) to compare estimated IAP-associated DALY losses calculated in the present study with estimates for these three established indoor health hazards.

To estimate the health impact from radon, SHS, and acute CO, we used Equation 2 with disease incidence estimates from the literature, summarized in Table 4. For radon and acute CO poisoning, only the end point of premature death was used to estimate DALY losses. The DALYs lost per incidence of various SHS outcomes and per early mortality due to acute CO poisoning and radon were taken from the literature and are also summarized in Table 4.

**Table 4:** Health outcomes attributable to SHS, radon, and acute CO poisoning in the United States and the DALYs lost per incidence of each health outcome.

Outcome	Annual U.S. excess incidence	DALYs lost per incidence
SHS				
Asthma episodes		202,300 (CalEPA 2005)		40/1,000 cases (Lvovsky et al. 2000)
Otitis media visits		790,000 (CalEPA 2005)		22/1,000 cases (de Hollander et al. 1999)
Sudden infant death syndrome		430 (CalEPA 2005)		78 (current U.S. life expectancy)/case (Xu et al. 2010)
Cardiac death		46,000 (95% CI: 22,700, 69,600) (CalEPA 2005)		1/case (de Hollander et al. 1999)
Lung cancer death		3,400 (CalEPA 2005)		14/case (de Hollander et al. 1999; Melse et al. 2010)
Radon lung cancer deaths				
Smokers		18,000 (95% CI: 5,600, 58,000) (U.S. EPA 2003)		14/case (de Hollander et al. 1999; Melse et al. 2010)
Nonsmokers		3,000 (95% CI: 950, 96,000) (U.S. EPA 2003)		14/case (de Hollander et al. 1999; Melse et al. 2010)
CO acute poisoning deaths		1.53 deaths per million persons (95% CI: 1.47, 1.59) (Centers for Disease Control and Prevention 2007)		32 (Xu et al. 2010)

*Comparison with DALY losses estimated by other methods.* Results from this study were compared with three other estimates of populationwide DALY losses for the United States. Although our study used an impact assessment approach, the studies used for comparison are cumulative risk assessment (CRA) and burden of disease studies (Ezzati and Lopez 2004; McKenna et al. 2005; WHO 2009). The burden of disease studies used available statistics to determine the disease incidence rate as a function of age, sex, and geographical location. A DALY value was then assigned based on YLL and disability incurred. The CRA studies determined the fraction of disease or death attributable to a specific risk factor based on epidemiological studies of specific populations. This is similar to, but far more complex than, our method of estimating health impacts due to SHS and radon. If the disease rate and DALY factors were accurate, and if we used the same discount ratings and time weightings for the age at which years of life are lost, both methods should estimate the same number of DALYs lost associated with a specific risk factor. Indoor air, independent of the impact of household use of solid fuels, had not been studied in a CRA analysis thus far. We compared results from our methodology with CRA results with the caveat that the methods are far from equivalent and the comparison should be seen only as a point of reference. The comparison also provides a useful tool for bounding uncertainties for our impact assessment method.

The WHO compiled disease incidence data for all communicable and noncommunicable diseases and injuries to determine the total number of DALYs lost per year for 192 countries (WHO 2009). McKenna et al. (2005) aggregated U.S. mortality and morbidity data to determine the top 20 causes of DALY losses for men and women in 1996. Ezzati and Lopez (2004) estimated the total DALYs lost due to smoking and tobacco use in industrialized nations by determining the impact of disease beyond what would be expected in nonsmoking homes. The total DALY losses that we estimated for all IAPs analyzed with the IND and ID methods were compared with estimates from these studies to discern whether the full CI of the aggregate IAP impact of indoor residential air is plausible. Additionally, we used our IND and ID methodology to calculate health impact for a suite of measured SHS components, and we compared the aggregate CI of the DALYs lost for these components with CRA-derived DALY estimates.

SHS is a complex mixture of chemicals. Nazaroff and Singer (2004) estimated increases in specific volatile organic compound concentrations (1,3-butadiene, 2-butanone, acetaldehyde, acetonitrile, acrolein, acrylonitrile, benzene, ethyl benzene, formaldehyde, naphthalene, phenol, styrene, toluene, and xylenes) expected for average smoking activity. Simons et al. (2007) found that homes with smokers had PM_2.5_ concentrations that averaged 16 µg/m^3^ higher than those in the homes of nonsmokers. We applied the IND and ID modeling frameworks established here to determine the additional DALYs lost due to living in a household that had indoor concentrations elevated by the specified levels. We used the Monte Carlo sampling to determine an aggregate CI for the DALYs lost due to exposure to this chemical mixture.

## Results

**Figure 1:**
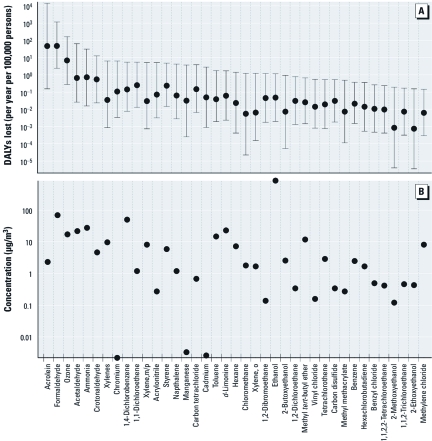
DALY losses associated with intake of indoor air (*A*), and mean chronic exposure concentrations (*B*), calculated with the ID model. The dots in *A* represent the central estimate of the DALYs lost, and the whiskers indicate the 95% CI.

Figure 1 shows the estimated number of DALYs lost due to indoor inhalation intake of HAPs and ozone based on the ID approach. Formaldehyde and acrolein had the largest estimated number of DALYs lost, 46 (95% CI: 0.2, 14,000) and 47 (95% CI: 2.4, 1,050), respectively, higher than the upper bound of the CI for all but two other pollutants, ozone and acetaldehyde. Of the 65 pollutants compared using the ID method, only 15 had 95% CIs that overlapped with the 95% CI for formaldehyde.

**Figure 2:**
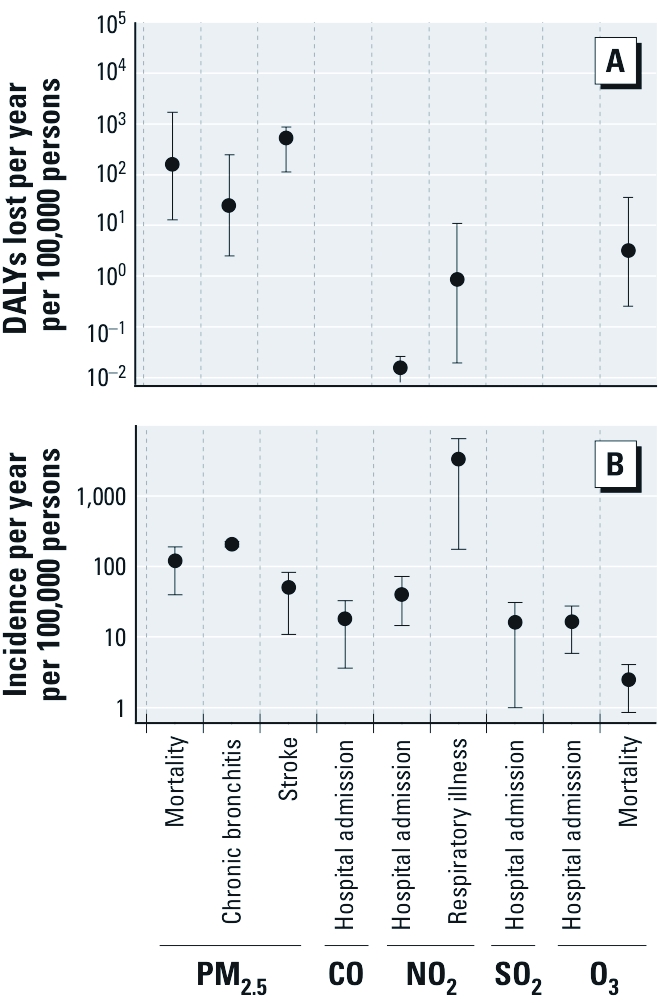
Annual DALYs lost (*A*) and incidence of disease (*B*; estimated by C-R functions) due to criteria air pollutant intake in residences, using the IND approach.

Figure 2 plots disease incidence and DALYs lost using the IND method for chronic exposure to criteria pollutants. The estimated DALYs lost because of incidences of stroke, chronic bronchitis, and premature death due to PM_2.5_ contributed substantially to annual health impacts. Mortality due to ozone is also a significant contributor to the total DALYs lost. The estimated number of DALY losses associated with hospitalization was relatively low for each pollutant. NO_2_ is potentially a significant acute health hazard, but we did not consider acute effects in this analysis. Nonlethal chronic exposure to CO and SO_2_ are not substantial contributors to DALYs lost from the outcomes we evaluated. There is concern that indoor concentrations of CO may have an adverse effect on certain susceptible populations (U.S. EPA 2010); however, the current empirical evidence is insufficient to reliably quantify the health impact from chronic CO exposure.

**Figure 3:**
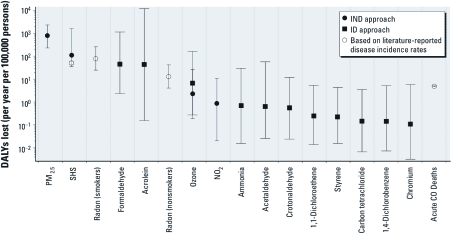
Estimated population-averaged annual cost, in DALYs lost, of chronic air pollutant inhalation in U.S. residences: results for the 12 pollutants with highest median DALY loss estimates. The markers represent the central estimate, and the whiskers indicate the 95% CI. Squares indicate pollutant DALY losses calculated using the ID approach. Circles indicate DALY losses calculated using the IND approach. Radon, acute CO deaths, and SHS DALY losses were calculated using disease incidence rates attributed to them in the literature.

Figure 3 shows the estimated DALYs lost from exposure to the 12 analyzed IAPs with the highest DALY losses per year per 100,000 persons. Figure 3 also shows estimates of DALYs lost per 100,000 persons per year attributed to SHS [51 (95% CI: 42, 60)], acute CO deaths [4.9 (95% CI: 4.7, 5.1)], and radon exposure for smokers [79 (95% CI: 25, 255)] and nonsmokers [13 (95% CI: 4, 42)]. For smokers, we overestimated the DALY losses attributable to radon per se because a portion of the DALY losses for smokers exposed to radon would result solely from smoking. For ozone, the IND and ID approaches estimated annual DALY losses per 100,000 persons of 6.7 (95% CI: 0.3, 160) and 2.3 (95% CI: 0.2, 26), respectively. There is substantial overlap in the CIs for both approaches, although the IND CI is smaller. These results suggest that PM_2.5_, acrolein, formaldehyde, radon, and SHS are the most harmful nonbiological air pollutants in residences on a population basis. In addition, we calculated that intake of the subset of compounds in SHS noted above would cause an annual loss of 1,000 DALYs (95% CI: 300, 14,000) per 100,000 residents in households with SHS, and a population-averaged annual loss of 110 DALYs (95% CI: 40, 1,600) per 100,000 residents in all U.S. households.

Our analysis yielded a central estimate for the DALYs lost due to all IAPs analyzed using the IND and ID methods of 1,100 DALYs per 100,000 persons (95% CI: 400, 13,000) per year. For 80% of the Monte Carlo samples, indoor PM_2.5_ was associated with the largest number of DALY losses, whereas acrolein and formaldehyde were the dominant contributors for 16% and 4% of the samples, respectively, and another IAP was the dominant contributor other than these three in < 0.25% of the Monte Carlo samples. For 90% of the samples, acrolein, formaldehyde, and PM_2.5_ contributed > 80% of the total DALYs lost. This reinforces the finding that these three pollutants account for most chronic health effects associated with indoor air in nonsmoking homes.

## Discussion

Although there is large uncertainty in the number of DALY losses estimated for each pollutant by the IND and ID models, several clear findings emerge. Our analysis demonstrates that in most U.S. residences PM_2.5_, acrolein, and formaldehyde dominate health impacts due to chronic exposures to nonbiological air pollutants. The DALY losses from these three pollutants appear to be much larger than the DALY losses due to CO deaths from acute poisoning in homes. SHS and radon are also significant contributors to populationwide DALY losses, but these exposures occur in a smaller fraction of homes.

Formaldehyde is primarily emitted from materials throughout the home. Similarly, acrolein is also emitted from such materials; however, cooking is also a potentially significant indoor source (Seaman et al. 2007). PM_2.5_ concentrations indoors, unlike acrolein and formaldehyde, are due to both indoor and outdoor sources, and outdoor concentrations may exceed indoor levels in many locations (Weisel et al. 2005).

Our analysis yielded a central estimate of 1,100 DALY losses per 100,000 persons (95% CI: 400, 13,000) per year for IAPs, excluding radon and SHS. For the United States overall, WHO (2009) estimated a total burden of 7,700 DALY losses per year per 100,000 persons for all noncommunicable, nonpsychiatric diseases combined. McKenna et al. (2005) identified the top 20 diseases that drive the health burden in the United States. Of those top 20 diseases, those with an indoor-air connection account for the loss of 3,000 DALYs per 100,000 persons per year (McKenna et al. 2005). Ezzati and Lopez (2004) estimated that the population-average burden of both firsthand (smokers) and secondhand tobacco smoke in industrialized nations is 12% of the annual DALYs lost, which we assume would represent 1,700 DALY losses per 100,000 persons per year, that is, 12% of the total DALY losses estimated for the United States by WHO (2009). Estimated DALY losses due to indoor PM_2.5_, acrolein, and formaldehyde combined [1,100 (95% CI: 700, 13,000)] were substantially greater than DALY losses due to the remaining 67 IAPs analyzed using the IND and ID methods combined [40 (95% CI: 10, 70)].

Our estimate of DALYs lost to SHS components in the 11% of homes estimated to have SHS is of similar magnitude to the mean estimate of DALY losses from IAP inhalation in nonsmoking homes. For the SHS analysis, pollutants that contribute the most to DALY losses are again PM_2.5_ and acrolein. Having a smoker in a residence, on average, doubles the concentrations of these two components relative to homes without smokers (Nazaroff and Singer 2004; Simons et al. 2007), effectively doubling the DALY loss estimates. The complete chemical mixture of SHS should be more toxic than the limited subset of components examined here, but the DALY loss estimate derived from the literature-reported health end points due to SHS is in the lower bound of the 95% CI. This result suggests that the component-based method used in this study may tend to overestimate DALY losses or that an insufficient number of health end points are attributed to SHS.

Both the IND and ID approaches rely on no-threshold disease incidence models that are linear or effectively linear over the concentration range of the analysis. The health impacts of PM_2.5_ are broadly thought to be linear at low doses (Schwartz et al. 2002). Threshold effects may significantly reduce the actual health impacts due to formaldehyde (a cancer hazard) and acrolein exposure (a noncancer hazard). Although some studies have identified genotoxic effects for formaldehyde (Viegas et al. 2010), others have identified strong threshold effects (Salthammer and Bahadir 2009). Various thresholds for exposure to avoid cancer have been suggested, ranging from 120 µg/m^3^ suggested by the German Federal Institute for Risk Assessment and WHO to 12 µg/m^3^ suggested by Naya and Nakanishi (2005). Given the distribution of formaldehyde concentrations determined for residences, threshold effect levels of 12 µg/m^3^ and 120 µg/m^3^ would result in 32% and 87% reductions in predicted DALY losses, respectively. A high threshold would result in PM_2.5_ and acrolein being the prime indoor pollutants of concern and formaldehyde being of lesser importance.

Less information is available on threshold levels for acrolein exposure. We determined the impact on the DALY loss estimate for a threshold equal to the CalEPA noncancer reference exposure level (0.35 µg/m^3^). Given the determined distribution of acrolein concentrations indoors, this would result in a 20% reduction in DALY losses.

## Conclusion

Using the methodology established here, we estimated that the total annual health impact of IAP inhalation in U.S. residences, excluding radon and SHS, is 1,100 DALY losses per 100,000 persons (95% CI: 400, 13,000). The upper bound of the range was twice as high as the number of DALY losses due to all noncommunicable, nonpsychiatric diseases as estimated by WHO (2009) based on disease statistics. The upper bound of the CI estimated for the DALYs lost due to exposure to a subset of pollutants in SHS using the methodology was also implausibly high. The total annual DALYs lost due to IAPs is likely in the lower half of the calculated range, that is, between the central estimate (1,100) and lower bound of the 95% CI (400) in DALY losses per 100,000 persons.

Because the upper-bound CI for all IAP-related DALY losses is too high, the upper-bound for CIs of at least some of the individual pollutants included must also be too high. Because the vast majority of the total DALY losses were due to acrolein, PM_2.5_, and formaldehyde, further statistical analysis may be able to narrow the currently large CIs for impacts from these pollutants.
